# Refueling T cell memory in cystic fibrosis

**DOI:** 10.1093/ajrcmb/aanag060

**Published:** 2026-05-11

**Authors:** Michael Shlossman, Daniel P Cook

**Affiliations:** Division of Pulmonary, Critical Care, and Occupational Medicine, Department of Internal Medicine, University of Iowa Carver College of Medicine, Iowa City, IA, United States; Division of Pulmonary, Critical Care, and Occupational Medicine, Department of Internal Medicine, University of Iowa Carver College of Medicine, Iowa City, IA, United States; Interdisciplinary Graduate Program in Immunology, University of Iowa Carver College of Medicine, Iowa City, IA, United States

For decades, cystic fibrosis (CF) has been understood as a disease of epithelial ion transport, with dysfunction of the Cystic Fibrosis Transmembrane Conductance Regulator (CFTR) anion channel that alters airway mucus properties, impairs mucociliary clearance, and leads to chronic infection.^1^ As our understanding of CFTR biology has deepened, a more complex picture has emerged. CFTR is expressed not only in epithelia but also in immune cells, including macrophages, neutrophils, and lymphocytes.^2^ The recent discovery that pulmonary ionocytes, rare epithelial cells comprising only 1%-2% of the airway, express the highest levels of CFTR per cell highlights a changing understanding of which cells matter most in CF.^3^ Evidence suggests we may also need to ask a fundamental question: Is CF an intrinsic disease of immune cells?

T cell dysfunction contributes to chronic lung inflammation and impaired pathogen control in CF.^4^^,^^5^ CD4+ T helper cells orchestrate immune responses through cytokine production and recruitment of other immune cell types. Once activated, naive T cells shift toward glycolysis to meet the bioenergetic demands of rapid proliferation and cytokine production.^6^ Memory T cells, in contrast, rely on oxidative phosphorylation and fatty acid oxidation, maintaining metabolic flexibility that allows them to rapidly respond upon antigen re-encounter. Could CFTR, an anion channel intimately involved in cellular ion homeostasis, influence these metabolic programs?

In this issue of the *Journal*, Saavedra and colleagues provide evidence that CFTR can influence T cell metabolic programs, and that restoring CFTR function reprograms these maladaptive circuits.^7^ Using CITE-seq to profile circulating CD3+ T cells from 18 adults with CF before and after initiation of elexacaftor-tezacaftor-ivacaftor (ETI), the investigators demonstrate that CFTR modulation induces transcriptional reprogramming, with CD4+ memory T cells showing the greatest changes. The transcriptomic signature reveals upregulation of genes involved in CD4+ T cell metabolism (*PNP* and *MT1X*) and enrichment of pathways related to mitochondrial metabolism, including electron transport chain components and oxidative phosphorylation machinery. Simultaneously, genes associated with chromatin remodeling and inflammatory activation decline following ETI therapy. The metabolic phenotype of CF memory T cells, it seems, shifts toward enhanced oxidative capacity following CFTR restoration, potentially improving T cell readiness for activation upon pathogen re-encounter.

But is this metabolic shift a direct consequence of CFTR restoration in lymphocytes or a downstream effect of improved clinical status? The authors address this question through complementary approaches. Using Seahorse metabolic flux analysis, they demonstrated that CF lymphocytes have reduced oxygen consumption and glycolytic capacity compared to healthy controls. Splenocytes from CFTR F508del homozygous mice recapitulate these findings, showing impaired oxidative and glycolytic metabolism with reduced ATP production compared to wild-type controls. CRISPR-Cas9 knockdown of CFTR in primary human lymphocytes reproduces the metabolic deficits, providing direct evidence that CFTR itself regulates lymphocyte bioenergetics independent of CF related-inflammation. If a chloride/bicarbonate channel can modulate mitochondrial function in immune cells, we may need to reconsider how ion transport and cellular bioenergetics are integrated.

Does metabolic reprogramming in CF T cells matter clinically? The correlations with improved patient outcomes suggest it might. Using linear mixed models, the authors identified that changes in CD4+ memory T cell gene expression are strongly associated with both FEV_1_ improvement and reduced hospitalizations for pulmonary exacerbations. Genes encoding electron transport chain complexes, including *NDUFA7*, *NDUFA13*, and *UQCRQ*, are upregulated in the strongest FEV_1_ responders. Plasma metabolomics revealed that circulating metabolites associated with mitochondrial function, including TCA cycle intermediates and acylcarnitines, correlate with both T cell gene expression changes and clinical outcomes.

These findings are provocative, but important caveats remain. The cohort of 18 subjects is small and may not capture the full heterogeneity of responses to ETI. The study design cannot distinguish whether lymphocyte metabolic changes fully result directly from CFTR restoration in lymphocytes versus indirect reductions in infection burden. While the CRISPR knockdown experiments suggest a direct role for CFTR, the relative contribution of cell-intrinsic versus environmental factors remains unclear. Additionally, whether these transcriptional changes translate to improved functional memory responses upon pathogen re-challenge requires direct assessment.

Despite these limitations, this work makes several important contributions. First, these data build on prior work demonstrating immunological effects of ETI,^8^^,^^9^ and establishes that CFTR modulation induces substantial transcriptional reprogramming in circulating T cells. Second, it identifies the metabolic fitness of memory T cells as a previously unrecognized target of CFTR function, extending the known roles of CFTR in mitochondrial biology from epithelia to immune cells.^10^ Third, it provides a mechanistic framework for understanding how CFTR restoration might improve host defense against chronic infection beyond increasing mucociliary clearance. Finally, the correlation between metabolic gene expression and clinical outcomes suggests that T cell metabolism could serve as a biomarker for treatment response.

T cells are implicated in CF lung inflammation, with exaggerated Th17 and Th2 responses and impaired regulatory T cell function contributing to tissue damage without improving pathogen control.^2^ We have largely attributed these abnormalities to the inflammatory environment of the CF lung. But what if the T cell dysfunction is, at least in part, cell-intrinsic? What if faulty CFTR renders T cells metabolically unfit for the demands of memory formation and recall? This would represent a paradigm shift, from viewing CF immune dysfunction as a consequence of chronic infection to recognizing it as a primary contributor to disease pathogenesis. CFTR modulators are being explored for non-CF conditions, including chronic bronchitis and COPD, where CFTR dysfunction is implicated in disease pathogenesis.^11^ If CFTR modulation can “reprogram” immune cell metabolism, might these drugs serve as a new class of immunometabolic modulators?

The work by Saavedra and colleagues reminds us that CF, despite decades of study, continues to yield surprises. ETI produces dramatic reductions in pulmonary exacerbations in part through improved mucociliary clearance. But perhaps T cell memory is also getting a metabolic fuel change, one that allows the adaptive immune system to finally contribute to, rather than simply perpetuate, the response to chronic infection. As CF care increasingly focuses on long-term management in an aging population, understanding these immunological consequences will be essential. For now, it seems that fixing CFTR may also mean refueling the immune system.

## Supplementary material

Supplementary material is available at *American Journal of Respiratory Cell and Molecular Biology* online.

## Supplementary Material

aanag060_Supplementary_Data
